# Sulforaphane: Its “Coming of Age” as a Clinically Relevant Nutraceutical in the Prevention and Treatment of Chronic Disease

**DOI:** 10.1155/2019/2716870

**Published:** 2019-10-14

**Authors:** Christine A. Houghton

**Affiliations:** ^1^University of Queensland, St Lucia Queensland, Australia; ^2^Cell-Logic, Australia

## Abstract

A growing awareness of the mechanisms by which phytochemicals can influence upstream endogenous cellular defence processes has led to intensified research into their potential relevance in the prevention and treatment of disease. Pharmaceutical medicine has historically looked to plants as sources of the starting materials for drug development; however, the focus of nutraceutical medicine is to retain the plant bioactive in as close to its native state as possible. As a consequence, the potency of a nutraceutical concentrate or an extract may be lower than required for significant gene expression. The molecular structure of bioactive phytochemicals to a large extent determines the molecule's bioavailability. Polyphenols are abundant in dietary phytochemicals, and extensive in vitro research has established many of the signalling mechanisms involved in favourably modulating human biochemical pathways. Such pathways are associated with core processes such as redox modulation and immune modulation for infection control and for downregulating the synthesis of inflammatory cytokines. Although the relationship between oxidative stress and chronic disease continues to be affirmed, direct-acting antioxidants such as vitamins A, C, and E, beta-carotene, and others have not yielded the expected preventive or therapeutic responses, even though several large meta-analyses have sought to evaluate the potential benefit of such supplements. Because polyphenols exhibit poor bioavailability, few of their impressive in vitro findings have been replicated in vivo. SFN, an aliphatic isothiocyanate, emerges as a phytochemical with comparatively high bioavailability. A number of clinical trials have demonstrated its ability to produce favourable outcomes in conditions for which there are few satisfactory pharmaceutical solutions, foreshadowing the potential for SFN as a clinically relevant nutraceutical. Although myrosinase-inert broccoli sprout extracts are widely available, there now exist myrosinase-active broccoli sprout supplements that yield sufficient SFN to match the doses used in clinical trials.

## 1. Introduction

We live in an era where modern medicine is strongly focused on relief of symptoms with pharmaceuticals, providing many solutions to address this demand. It is becoming increasingly apparent, however, that for the diseases which cause most distress at the individual level, pharmaceuticals typically provide only short-lived symptomatic relief. Few if any modern pharmaceuticals modulate fundamental etiological disease processes.

As a consequence, there is a groundswell of interest in phytochemical solutions which may potentially target the fundamental *upstream* causes of disease [1, 2]. Plant-derived bioactive compounds are already emerging as candidate molecules with significant therapeutic potential in human health [3]. Numerous mechanistic investigations of phytochemical bioactives are already helping to elucidate the pathophysiology of both chronic diseases and acute self-limiting conditions [4]. It is generally considered that such findings may inform the development of new therapeutic solutions. Although pharmaceutical medicine has historically looked to plants as sources of the starting materials for drug development, the ultimate therapeutic molecule is typically quite different from the original plant-derived source. By contrast, the focus of nutraceutical medicines is to retain the plant bioactive in as close to its native state as possible. The challenge for developers of nutraceutical supplements is that the potency of such nutraceutical concentrates or extracts may be below the threshold required to nutrigenomically induce the gene expression required for a significant therapeutic response.

### 1.1. Searching for Upstream Factors

Because homeostasis in human cells is reliant on the dynamic integration of many core biochemical processes, a search for *upstream* factors in the etiological processes of disease is the focus of considerable global research; such research is closely focused on investigating signalling pathways within cells and organelles. Prior to the introduction of better hygiene practices, the global disease burden was dominated by infectious diseases. By contrast, more recent decades have seen a steady increase in levels of morbidity and mortality rates from chronic disease, justifying the claim that chronic disease has reached epidemic proportions [5].

As one example, the increasing global prevalence of cardiovascular disease (CVD) and type 2 diabetes (T2DM) is dominant in the current trajectory for chronic disease. It is emerging [6] that the primary upstream factor which links endothelial dysfunction with CVD and T2DM and described as cardiometabolic disease is closely related to oxidative stress [6–11].

More recently, mechanistic studies link cardiometabolic dysfunction with intestinal dysfunction and subsequent metabolic endotoxaemia. The cell walls of gram-negative bacteria increase luminal levels of lipopolysaccharides (LPS) that are detected by and bind to Toll-like receptor 4 (TLR4). This initiates the activation of Nf-*κ*B with the subsequent generation of inflammatory cytokines that are systemically absorbed [12]. At least three apparently distinct mechanisms—endoplasmic reticulum stress, toll-like receptor (TLR) 4 activation, and changes in gut microbiota—have been identified as triggers of obesity-associated metabolic inflammation [13]. SFN, the focus of this review, has been identified as a molecule that can reduce inflammation via inhibition of LPS-TLR4 binding [14, 15]; this mechanism is further discussed in Section 7.5.

### 1.2. Failed Antioxidant Trials

Attempts to use the classical antioxidant vitamins to enhance endothelial function and related glucose modulation have largely resulted in no response in some studies and adverse effects in others [9, 16–19].

A 2010 meta-analysis [20] of major randomized placebo-controlled trials (98,886 subjects in total, Table 1) investigating the effects of the antioxidant supplement on prevention of diabetes or effect on glucose homeostasis showed no effect from vitamin E, vitamin C, beta-carotene, selenium, zinc, and combinations of these.

Similar meta-analyses also fail to demonstrate significant chemoprotection or preventive benefits against cancer and cardiovascular disease via antioxidant vitamins [16, 21–23]. These findings suggest the possibility that intervention with phytochemicals as redox-modulating biomolecules might provide an alternative but effective strategy.

### 1.3. SFN and Type 2 Diabetes Intervention Trials

Whereas Table 1 lists large-scale clinical trials considering T2DM risk in thousands of individuals over long periods, the studies in which SFN has been utilised as the intervention material are few, are of short duration, and include small numbers of participants.

To query whether SFN as an indirect antioxidant could modify disease risk in T2DM where direct-acting antioxidants seemed unable, a 4-week randomized controlled clinical trial [24] was conducted in 2011 to investigate the effect of 5 grams (yielding 112.5 *μ*mol SFN) and 10 grams (yielding 225 *μ*mol SFN) daily of broccoli sprout powder on 81 T2DM patients and using cardiometabolic biomarkers as the outcome measures. The results provided the first data to show that SFN could reduce lipid peroxidation, especially significant at the higher dose. In particular, favourable redox status was demonstrated by a decrease in plasma malondialdehyde (MDA) and oxidised LDL (OX-LDL). As increased lipid peroxidation in diabetes has been implicated as an important factor in the pathogenesis of T2DM complications, the researchers considered the potential for SFN to play a role in the prevention of T2DM and its secondary effects.

In general, the studies which have used SFN-yielding broccoli sprouts or supplements to enhance cellular defences have shown promising mechanistic findings but inconsistent clinical responses [25–30]. A 2018 study investigating biomarkers of inflammation in overweight but otherwise healthy adults showed significant downregulation of two such biomarkers; this is expanded in Section 7.6 with the clinical trial data discussed in Section 7.7. These data hold promise for the clinical application of SFN in inflammation-related conditions.

### 1.4. The Origin of Antioxidant Supplements as Therapy

The notion that aging was due to a state of oxidative stress within cells emerged in the 1950s from Dr. Denham Harman, a research chemist in the petrochemical industry who subsequently joined the faculty of the University of Nebraska Medical Center. His “*free radical theory of aging*” postulates that the typical changes that occur during aging are caused by free radical reactions [31].

The theory gained initial support by others including Nobel laureate, Linus Pauling whose hypotheses on ascorbic acid deficiency as an etiological factor in cancer and acute infectious illnesses earned him enormous popularity with consumers but derision within much of the scientific community [32]. It could be argued that the promotion of vitamin C as a “cure” for the common cold and for cancer heralded the onset of a huge upsurge in sales of antioxidant vitamins.

It would seem that because plant-based diets had been shown epidemiologically to be protective against a range of diseases [33], it had been erroneously assumed that the protective effect was conferred by the presence of vitamins like A, C and E and beta-carotene. Had these early researchers also considered that plant foods are endowed with an extensive range of bioactive phytochemicals functioning via different mechanisms, they may not have drawn this conclusion [34–36].

### 1.5. Addressing a More Nuanced View of Redox Balance

The study of the relationship between oxidative stress, aging, and disease remains popular, with investigators striving to identify interventions that are capable of modulating the disease-causing processes [37]. The free radical-antioxidant theory proposed decades ago proved to be too simplistic; more recent research reveals multiple signalling processes at play [8]. As we observe the unfolding of the complex relationships governing endogenous cellular mechanisms, a close interconnectedness between redox balance, inflammation, and endoplasmic reticulum stress emerges [38]. What this indicates is that any therapeutic attempt to successfully intervene must either address each process individually or intervene sufficiently *upstream* at a point that can beneficially influence multiple *downstream* targets.

It is within this framework of redox-associated disease that this review considers mechanisms by which the Brassica-derived phytochemical, sulforaphane (SFN), may be utilised therapeutically to modulate the *upstream* cellular perturbations that contribute to the etiology of disease.

A number of large systematic reviews and meta-analyses, including Cochrane Reviews, have concluded that although oxidative stress underpins common chronic diseases, antioxidant vitamins do not lead to reduction in disease risk [16, 22, 39–46].

## 2. Phytochemicals as Inducers of Endogenous Defences

A possible alternative approach to the modulation of oxidative stress by direct-acting antioxidant vitamins involves the application of phytochemicals with nutrigenomic potential [47]. By definition, a phytochemical is a plant-derived chemical substance that is biologically active but typically nonnutritive [48]; nutrigenomics describes the way in which phytochemicals and nutrients may affect gene expression. As such, the application of nutrigenomic principles may allow effective dietary intervention strategies to recover normal homeostasis and to prevent or even treat diet-related diseases [49]. Phytochemicals are abundant in the food supply and have been classified according to their molecular structure, a property which contributes to their observed beneficial on human health [36, 50].

### 2.1. Polyphenols: Their Clinical Potential

Polyphenolic bioactives derived from plant species have been extensively researched in relation to their mechanisms of action in human cells and for their clinical potential in modulating disease-causing processes [51]. Such molecules have significant *in vitro* antioxidant potential, but their low bioavailability [52, 53] limits their clinical usefulness as systemic antioxidants.

Consequently, although extensively studied *in vitro,* attempts to replicate these findings *in vivo* have been disappointing and it is generally considered that the large molecular weight and structure of these molecules is a significant factor impeding their bioavailability [50, 54]. Another role for polyphenols has more recently been identified in relation to their prebiotic and other beneficial effects on the gut microbiota [55].

A very recent meta-analysis of clinical trials in which polyphenol-based supplements were evaluated for their beneficial effects on specific markers of cardiovascular risk and cognitive status concluded that definitive recommendations for the use of these compounds could not yet be made and that additional characterisation of pharmacokinetics and safety is required [56]. The bioavailability of the polyphenolic phytochemicals so abundant in the food supply lies between 1% and 10%. This is discussed in some depth in Section 6 of an earlier review by this author [54].

**Figure 1:**
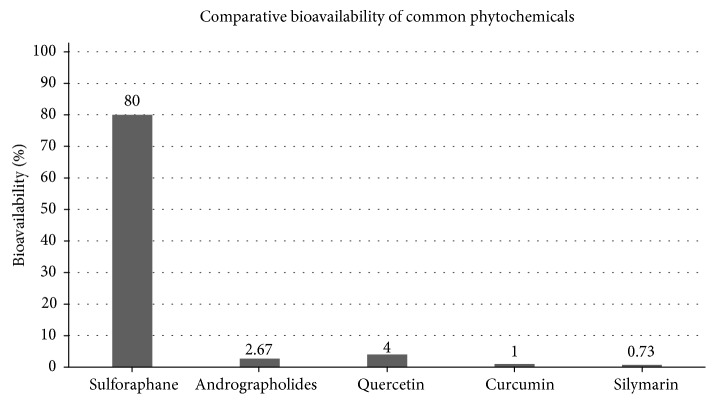
Comparative bioavailability of phytochemicals commonly used in dietary supplements (appears as Figure 3 in [54]).

SFN, derived primarily from broccoli and with absolute bioavailability of around 80% [57], shows promise as a nutrigenomically active compound capable of increasing several endogenous antioxidant compounds via the transcription factor, Nrf2 [58]. SFN, an aliphatic isothiocyanate [59], emerges as a phytochemical with comparatively high bioavailability due to its low molecular weight and log*P* value of 0.23 [60] when evaluated using the commonly used octan-1-ol and water system and where log*P* = 0 represents equal partitioning between the solvent and water and where a value > 0 represents a higher concentration in the lipid phase [61].  Figure 1 highlights the differences in bioavailability when comparing SFN with polyphenols commonly used in dietary supplements.

### 2.2. The Nutrigenomic Link to Endogenous Antioxidants

Nutrigenomically active phytochemicals exhibit a number of different mechanisms as modulators of the expression of genes coding for enzymes which are active in diverse pathways [62]. One of the intended effects of this strategy is to increase the production of *endogenous* antioxidant compounds including the antioxidant enzymes. Whilst some phytochemicals may upregulate cellular endogenous defences, others may downregulate pathways associated with undesirable prolonged inflammation. The key transcription factors responsible for the induction of redox-modulating and inflammation-promoting gene expression are, respectively, Nrf2 and NK-*κ*B; these transcription factors act both independently and cooperatively via cross talk that is not yet fully understood [63].

Although such plant-derived compounds may exhibit direct antioxidant activity, it is their *indirect* antioxidant effect which is of most interest, due to the catalytic effect of the antioxidant enzymes in quenching reactive oxygen and reactive nitrogen species (ROS and RNS) compared with nonenzyme antioxidants which exhibit only a one-for-one stoichiometric effect [64, 65]. There is considerable evidence to show that induction of such cytoprotective compounds has multiple beneficial effects [66–69].

Germinated broccoli seeds yield a nutrigenomically active isothiocyanate, SFN; this review focuses on the properties of SFN as they relate to its antioxidant, anti-inflammatory, and antimicrobial effects. Furthermore, this discussion reviews the doses used in relevant clinical trials with a view to evaluating whether these doses are practical for SFN to be considered as a nutraceutical with broad clinical application and whether it may be considered as an efficacious nutraceutical in the prevention and treatment of chronic disease.

### 2.3. Determining Clinical Potential of a Phytochemical

When considering the likelihood that a particular phytochemical may exhibit clinical potential, two important factors bear mention. Firstly, the bioactive molecule must have sufficient potency to induce adequate gene expression in the target gene or genes; secondly, the bioactive must be sufficiently bioavailable that the concentration measurable in the bloodstream or target tissue is able to match the concentrations measured in the *in vivo* studies for which gene expression is significant [54].

It is not uncommon for *in vitro* concentrations to yield impressive changes in gene expression, but this is of no practical value if the compound exhibits poor bioavailability. Polyphenols commonly fall into this category, with bioavailability preventing the *in vivo* replication of *in vitro* outcomes when the same molecule is ingested in an oral dose form [70–72].

### 2.4. The History and Evolution of Sulforaphane Research

**Figure 2:**
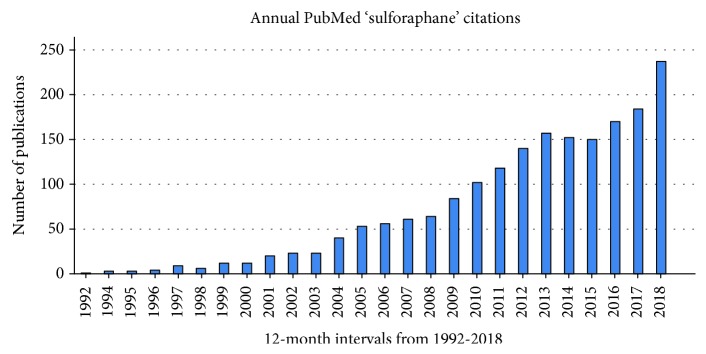
Sulforaphane research timeline; PubMed.

It is twenty-five years since the identification and isolation of the transcription factor, Nrf2 (coded by the gene *nuclear factor erythroid 2-related factor 2*), was first described in the scientific literature [73]. In the ensuing years, Nrf2 has become a focus of active research on mechanisms of defence in mammalian cells; Figure 2 illustrates the upward trend in SFN research over the period [74]. The role of Nrf2 in human cells is very relevant to the subject matter of this review because SFN significantly activates Nrf2 and as such has the potential to modulate the expression of genes associated with redox balance, inflammation, detoxification, and antimicrobial capacity, all key components of the *upstream* cellular defence processes [75].

There are many factors that can activate Nrf2. In addition to diet-derived molecules, a range of environmental stressors function as signals to activate Nrf2 and consequent expression of a battery of defensive genes [76]. Numerous commonly ingested phytochemicals are Nrf2 activators, and the reader is referred to a detailed discussion of the chemical properties and the subtle differences of individual phytochemical Nrf2 activators in relation to their interactions within relevant biochemical pathways in human cells [77].

In addition to providing a list of the more extensively studied phytochemical Nrf2 activators, Eggler and Savinov suggest, in their concluding remarks, that although it is unlikely that a single phytochemical will emerge as a magic bullet for disease prevention or amelioration, future prospects could include phytochemical “cocktails” formulated for their synergistic effects [77]. In this regard, a larger quantity of low potency Nrf2-activating phytochemicals may provide the same effect as smaller quantities of a single Nrf2 activator such as SFN. If an additive or a synergistic effect of multiple Nrf2 activators provides significant Nrf2 activation, it may explain why diets rich in plant foods have been shown epidemiologically to significantly benefit human health [78].

Interest in SFN as a food-derived compound with significant clinical potential began in 1992 when a group [79] at Johns Hopkins University published its findings. The group had published two papers to support their research on the induction of anticarcinogenic enzymes derived from broccoli and on assay methods to rapidly detect such enzymes [79, 80]. Interestingly, SFN was identified here as a potent activator of cellular defence mechanisms approximately two years before the isolation of Nrf2 by Moi et al. [73] and Zhang et al. [79].

Broccoli-derived SFN was capable of activating the cytoplasmic transcription factor, Nrf2, which in turn translocated to the nucleus to activate the Antioxidant Response Element (ARE) in the promoter region of several hundred identified genes [58, 66, 81, 82]; many of which are related to cellular defence processes.

The Johns Hopkins group had found that the 3-day germinated broccoli seed contained 20-50 times more of the precursor glucoraphanin (GRN) than did the mature broccoli vegetable [81]. It was this finding that enabled the design of trials which could achieve clinically relevant SFN effects with small practical doses of dried broccoli sprouts.

## 3. Sulforaphane: Structure-Function Relationship

### 3.1. Physical Properties of Sulforaphane as an Intervention Compound

**Figure 3:**
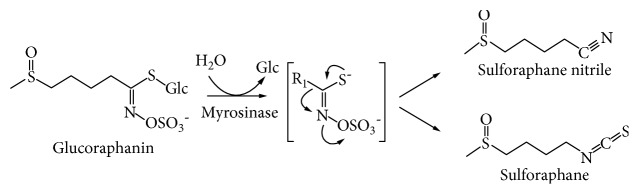
The synthesis of isothiocyanates via a hydrolysis reaction of the glucosinolate by the myrosinase enzyme. Sulforaphane is the isothiocyanate synthesised from the glucosinolate, glucoraphanin (an image adapted from Dinkova-Kostova et al. [86]).

SFN is naturally derived from certain species of the *Brassica* vegetable family [83], most notably broccoli. Classified as cruciferous vegetables, they are known for their disease-preventive effects [84, 85]. When ingested, the bioactivity of crucifers is dependent on the dual presence of a precursor molecule, a *glucosinolate*, and an enzyme, *myrosinase*, which hydrolyses the precursor; the product is an isothiocyanate (Figure 3) [86].

Broccoli has been shown to be the most significant dietary source [87] of the precursor glucosinolate, GRN, which, in the presence of the *myrosinase* enzyme, is metabolised to SFN. Young sprouted broccoli seeds in the order of 3-7 days' growth have been shown to contain the highest GRN levels [81].

**Figure 4:**
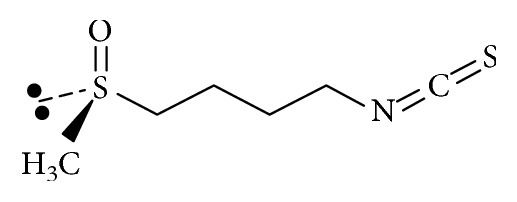
Sulforaphane (C_6_H_11_NOS_2_)—molecular structure of sulforaphane (4-methylsulfinylbutyl isothiocyanate).

The structure of this small molecule (M.W. 177.29 and log*P* = 0.23) confers upon SFN some unique advantages not afforded other phytochemicals such as the polyphenols which are structurally large and essentially hydrophilic [70]. One of the major advantages for SFN is its higher bioavailability, a consequence of its structure and lipophilicity (Figure 4).

### 3.2. Bioavailability: Relationship to Molecular Structure

SFN has been demonstrated to have an absolute bioavailability of around 80% [57] and to peak in the bloodstream around 1 hour following ingestion [88, 89]. By comparison, the polyphenols which are large bulky higher molecular weight molecular structures typically exhibit bioavailability of around 1-8% [90].

For a food-derived molecule to achieve sufficient intracellular concentration to affect gene expression, its bioavailability must be high enough that it can be absorbed through the intestinal epithelium and the several other membranes between the gut and the target cell. With its high bioavailability, SFN can therefore be considered as having at least one of the key properties necessary to be considered for development as a nutraceutical compound.

## 4. Sulforaphane as a Molecule with Nutrigenomic Properties

Nrf2 has been variously described by several researchers as an “*activator of cellular defence mechanisms*” [91], “*the master redox switch*” [92], and “*a guardian of health span and gatekeeper of species longevity*” [93]. As a mediator for amplification of the mammalian defence system against various stressors, Nrf2 sits at the interface between our prior understanding of oxidative stress and the endogenous mechanisms cells use to deal with it [54].

What is emerging is that diseases known to be underpinned by oxidative stress are proving to be more responsive to such amplification of cellular defences via Nrf2 activation than by administration of direct-acting antioxidant supplements [22, 94].

### 4.1. The SFN-Nrf2: Activating Gene Expression in Cellular Defences

**Figure 5:**
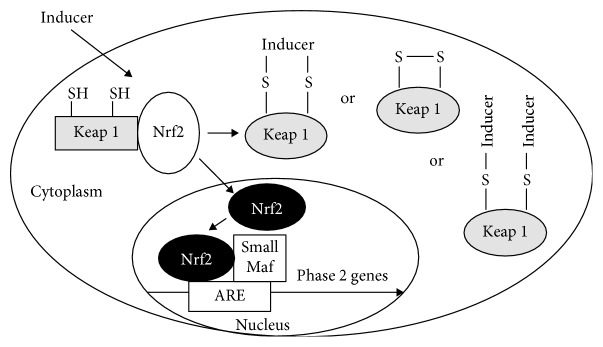
Mechanism by which an inducer affects expression of Phase 2 detoxification genes (an image from Zhang et al. [97]).

The essence of a very complex biochemical process [74] is that in its basal state, Nrf2 is sequestered to Kelch-like ECH-associated protein 1 (Keap-1) and associated with cytosolic actin filaments; however, when Keap-1 detects a stressor which may threaten the cell's integrity, activation of the complex leads to a dissociation of Nrf2 from Keap-1 [95]. Hereafter, it translocates to the nucleus where it may induce expression of its many target genes, aligning with the ARE in the promoter region of these genes. The ARE is a *cis*-acting enhancer sequence that is upstream of many Phase 2 detoxification and antioxidant genes [96] (Figure 5 [97]).

Loss of the Nrf2-ARE function in mice has been shown to increase susceptibility [98] to acute toxicity, inflammation, and carcinogenesis due to the inability to mount adaptive responses. The elucidation of this process showed that the activation of Nrf2-ARE induces a large battery of cytoprotective enzymes [99].

Cellular Nrf2 levels are under strict control by multiple mechanisms but the best-characterised is the one which is mediated by interaction with Keap-1 [63]. Keap-1 not only binds Nrf2 to cytoplasmic actin filaments in the basal state but it also acts as a sensor, especially of subtle redox changes in the cell.

The chemistry of sulfur plays an integral role in Nrf2 activation and subsequent modulation of gene expression. All Nrf2 activators react with thiol groups. Keap-1 is rich in sulfur-rich cysteine residues [99] and is under oxidation-reduction (and alkylation) control via its highly reactive thiol groups.

An inducer such as SFN activates the Nrf2-Keap-1 complex, with sulfur chemistry playing an important role [99].

### 4.2. The Significance of the Nrf2-SFN Relationship

Nrf2 is ubiquitously expressed with the highest concentrations (in descending order) in the kidney, muscle, lung, heart, liver, and brain [73, 100]. The activation of Nrf2 activators has been found to be closely associated with their molecular structure [100–102]. Because food-derived SFN is readily bioavailable, such universal Nrf2 tissue distribution enhances SFN's potential to modulate systemic gene expression [92].

The properties of Nrf2 are such that it can be considered a novel drug target with potential applications across a broad range of conditions. Interestingly, the Nrf2-activating properties of SFN have been experimentally used in conjunction with pharmaceuticals. By way of an example, SFN's effect on Nrf2 has been investigated in this context as a means of minimising the nephrotoxicity which typically limits the use of the chemotherapeutic drug, cisplatin [103]. Another example illustrates a synergistic antioxidant and anti-inflammatory response when SFN is combined with Exemestane, a synthetic steroidal inhibitor of the aromatase reaction that catalyses the terminal and rate-limiting step of the biosynthesis of estrogens. The combination may be considered to be protective against other chronic diseases unrelated to aromatase inhibition and the significance of such coadministration is expanded in Section 9.4 [104].

### 4.3. Pleiotropic Effects of SFN

Although SFN is most often considered for its Nrf2-dependent effects and largely associated with the induction of antioxidant and Phase 2 detoxification enzymes, other less well-characterised mechanisms are associated with this phytochemical molecule. These Nrf2-independent mechanisms include but are not limited to the induction of apoptotic pathways, suppression of cell cycle progression, inhibition of angiogenesis and anti-inflammatory activity, and inhibition of metastasis, primarily relevant to cancer [62].

One such effect is its action as a histone deacetylase (HDAC) inhibitor [105, 106], and there is a growing focus on the role of SFN and other phytochemicals on such epigenetic effects [107, 108] and more recently on the role of SFN as an inhibitor of microRNAs [109]. Epigenetic effects are of particular clinical interest in that such changes are potentially reversible and thereby may provide an opportunity for intervention in earlier stages of the cancer process [110]. Tumour suppressor genes such as p53 may be epigenetically inhibited [111] so that therapies aimed at removing such suppression are attractive options, especially if they can be available through dietary means.

No discussion of SFN and Nrf2 would be complete without reference to the fact that both Nrf2 activators and Nrf2 inhibitors can be utilised in cancer therapy. A very recent paper [112] highlights this dual role and its implications for Nrf2 activation. It suggests that because Nrf2 can modulate the detoxification pathways, its effect on anticancer drugs may lead to chemoresistance and that the switch between a beneficial and a detrimental role for Nrf2 in cancer cells depends on a number of factors which include the tight control of its activity. This poses an obvious dilemma which is already under active discussion and investigation [113–115]; SFN and other phytochemicals capable of modulating Nrf2 form part of such investigation [112].

A 2012 gene expression study to evaluate the effect of SFN as an Nrf2 activator showed that despite the very large 5- to 20-fold increase in Nrf2 binding at their AREs, only a small increase in expression signal was observed. The researchers concluded that there may be other determinants, such as tissue-specific cofactors, negative feedback loops, and epigenetic or signalling mechanisms, which affect both basal expression and Nrf2-mediated transcriptional regulation of these highly expressed genes in cells [116].

### 4.4. Major Actions of SFN at the Cellular Level

The major documented cellular actions of SFN are listed in the nonexhaustive summary shown in Table 2 along with commentary on their clinical implications. These *upstream* processes have significant *downstream* effects and are associated with the observed effects in clinical trials using SFN or a dietary source of SFN. Most but not all of these actions are associated with Nrf2 activation.

## 5. Sulforaphane in Core Cellular Processes

### 5.1. Multiple Gene Targets and the Nrf2/ARE Pathway

It has been suggested that well in excess of 500 genes have been identified as being activated by SFN via the Nrf2/ARE pathway [132–134], and it is likely that this underestimates the number as others are being discovered.

The large battery of upregulated cytoprotective genes includes those coding for the endogenous enzyme and nonenzyme antioxidants as well as Phase 2 detoxification enzymes [58]. Nrf2 plays a crucial role in the coordinated induction of those genes encoding many stress-responsive and cytoprotective enzymes and related proteins [135]. These include NAD(P)H:quinone reductase-1 (*NQO1*), haemoxygenase-1 (*HO-1*), glutamate-cysteine ligase (*GCL*), glutathione-S-transferase (*GST*), glutathione peroxidase (*GPX1*), thioredoxin (*TXN*), thioredoxin reductase (*TXNRD1*) [92], and PPAR-*γ* (*PPARG*) [136].

These endogenously-generated enzyme and nonenzyme molecules are not generally considered to necessarily function as “antioxidants” even though they exhibit significant redox-modulating capacity *as* and *when* the cell requires it.

When Zhang and colleagues [79] of the Johns Hopkins group were investigating chemoprevention in the early 1990s, they had been working on cytoprotective genes including those coding for the Phase 2 detoxification enzymes *NQO1* and the *GST* families; the discovery that these genes were significantly induced by broccoli sprout-derived SFN provided the foundation for the rapid interest in research in this field.

Of the available SFN clinical trials associated with genes induced via Nrf2 activation, many demonstrate a linear dose-response (Table 3). More recently, it has become apparent that SFN can behave hormetically [137] with different effects responsive to different doses. This is in addition to its varying effects on different cell types and consequent to widely varying intracellular concentrations [125, 138–142].

### 5.2. SFN as a Redox Modulator

Even though enzymes known to function within the Phase 2 detoxification pathway are not typically considered to be “antioxidants,” it has now been firmly established that NQO1 provides major antioxidant functions by virtue of its obligatory two-electron reduction mechanism which diverts quinones from participating in oxidative cycling and generation of reactive oxygen intermediates.

**Figure 6:**
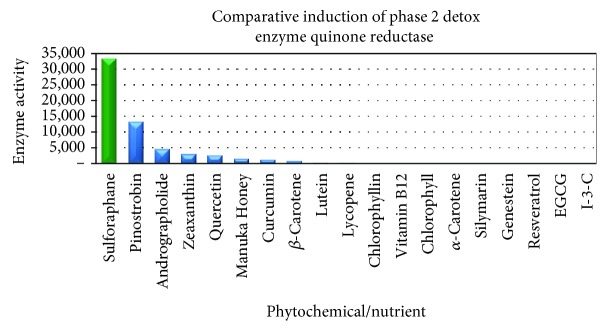
Comparison of capacity to induce NQO1 by a range of phytochemicals, indicating that SFN exhibits many-fold greater inducer ability (data compiled from Yang and Liu [168] and Fahey and Kensler, 2008).

A major new perspective on the functional importance of this enzyme [143–145] followed the finding that the gene coding for NQO1 is highly inducible and that its increased induction protected animals and their cells against oxidative stress [143–145]. SFN is considered to be one of most potent phytochemical inducers of *NQO1* [96, 146]. As such, SFN's nutrigenomic effects contribute to the enhancement of the cell's antioxidant capacity [64].  Figure 6 illustrates the comparative induction of SFN and other phytochemicals.

### 5.3. Endogenously Generated “Antioxidants” in Type 2 Diabetes

Given the role of SFN in induction of Nrf2-dependent cytoprotective genes, SFN might be a useful candidate for modulation of *upstream* genes associated with the etiology of T2DM. A 2016 review paper reaffirms a rationale for the “unifying hypothesis” proposed by Brownlee in 2001 in which generation of ROS is the key central theme linking the pathogenesis of T2DM and CVD [147]. In further support of this hypothesis, Rask-Madsen and King reinforce the possibility that endogenous protective pathways could protect against vascular complications in T2DM [148]. The following sections highlight the role of several inducible redox-modulating molecules with reference to their activity in T2DM.

### 5.4. Highlighting Redox-Modulating Nrf2 Target Genes

Several well-studied Nrf2-dependent target genes of possible relevance are those encoding synthesis of glutathione (GSH), Trx, HO-1, and NQO-1. Each has been shown to be induced by SFN in a variety of cell types, including endothelial cells. A study [149] using human aortic cells showed that the activation of the Nrf2-ARE pathway may represent a novel therapeutic approach for the treatment of inflammatory diseases such as atherosclerosis.

In support of this approach, a 2009 combined cell culture/animal study [150] showed that shear stress in blood vessels keeps Nrf2 in an activated state and as such protects against endothelial dysfunction. Activated by SFN, Nrf2 was shown to prevent endothelial cells from exhibiting a proinflammatory state via the suppression of p38-VCAM-1 signalling, providing a novel therapeutic strategy to prevent or reduce atherosclerosis.

In other tissues of the cardiovascular system, Nrf2 has been shown to regulate both basal and inducible ARE-controlled cytoprotective genes in cardiomyocytes [151]. As with endothelia, Nrf2 is required for protection against glucose-induced oxidative stress and cardiomyopathy in the heart.

## 6. SFN: Its Redox-Modulating Effects

### 6.1. Glutathione

The nonenzyme antioxidant GSH is a major contributor to cellular redox status and the rate-limiting enzyme for its synthesis; glutamate-cysteine ligase (coded by the gene *GCL*) can be induced by SFN [152]. Antioxidants in general and glutathione in particular can be depleted rapidly under conditions of oxidative stress, and this can signal inflammatory pathways associated with NF-*κ*B [153]. Nrf2 has been found to be the primary factor inducing the cell survival system under GSH depletion [154]. Also of interest is the finding that Nrf2 transcriptional activity declines with age [155, 156], leading to age-related GSH loss among other losses associated with Nrf2-activated genes. This effect has implications too for decline in vascular function with age [157].

Some of the age-related decline in function can be restored with Nrf2 activation by SFN [158]. Studies in aged mice showed that age-related changes in Th1 immunity could be restored using SFN as an intervention. This finding is compatible with the growing recognition of the importance of the Nrf2 pathway in innate immunity and has implications for human health [159]. A 2017 clinical pilot study examined the effect of an oral dose of 100 *μ*mol (17.3 mg) encapsulated SFN on GSH induction in humans over 7 days [158]. Pre- and postmeasurement of GSH in blood cells that included T cells, B cells, and NK cells showed an increase of 32%. Interestingly, the researchers found that in the pilot group of nine participants, age, sex, and race did not influence the outcome.

Disturbances of thiol-related mechanisms have been observed [160] in diabetes, with plasma levels of protein-bound thiols lower in T2DM than in controls. These thiols include GSH and Trx. An animal study [161] illustrates the relationship between depressed GSH and the development of atherosclerosis. In this experiment [7], the rate-limiting enzyme in GSH synthesis, *gamma-glutamyl-cysteine synthetase* (*γ*-GCS), was shown to be downregulated *early* in the atherosclerosis process. This effect preceded the appearance of lipid peroxidation products by several months. The antioxidant enzyme, glutathione peroxidase (GPx) was simultaneously downregulated.

Erythrocyte levels of GSH have been shown to change depending on the stage of the diabetic process of the individual [162]. It has been shown that compared to controls, prediabetic patients exhibit a significant lowering of GSH [163]. As the disease progresses to diabetes and later to diabetes with cardiovascular complications, GSH levels rise; however, they do not reach the levels of controls. The variability in GSH levels depending on the stage of the disease makes it difficult to use GSH as an effective clinical trial biomarker to measure change.

An infusion of GSH as an intervention in a clinical trial [164] was shown to reverse endothelial dysfunction by strongly potentiating the effect of acetylcholine-mediated vasodilation via enhanced nitric oxide activity. Because GSH as a tripeptide molecule is degraded by gastric proteolytic enzymes, it is generally considered as being unsuitable as an oral therapeutic [165]. If SFN can be shown to induce GSH in endothelial cells, this may provide an alternative means of enhancing GSH levels in endothelial and pancreatic beta-cells with a view to reducing the complications of T2DM together with the many conditions for which dysregulated GSH is associated.

### 6.2. Thioredoxin: Protection from Elevated Blood Glucose

Thioredoxin (Trx) is a potent protein disulfide that participates in many thiol-dependent cellular reductive processes and plays an important role in antioxidant defence, signal transduction, and regulation of cell growth and proliferation. As a cellular thiol, Trx has been shown [166] to be associated with the development of diabetic complications. Like GSH, Trx has been shown to protect cells against high ambient glucose [167].

**Figure 7:**
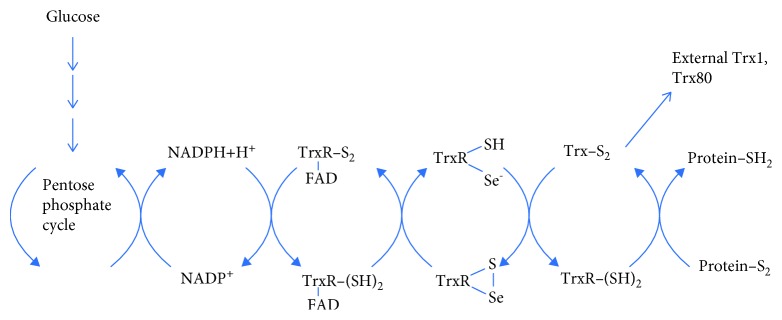
The thioredoxin system and its relationship with glucose metabolism in the pentose phosphate cycle. The pentose phosphate cycle generates reducing equivalents which are transferred along a series of cycling redox reactions. Induction of Trx and Trx reductase by SFN enables glucose to be metabolised as an alternative to the synthesis of superoxide radical to alleviate much of the metabolic stress associated with T2DM (a figure adapted from Holmgren and Lu [169]).

The thioredoxin system (Figure 7) consists of thioredoxin, thioredoxin reductase, and NAD(P)H.

Like GSH, Trx contributes to the cellular thiol pool [170] with the thioredoxin system shown to exhibit cardioprotective effects [171]. The pentose phosphate pathway can alleviate much of the oxidative stress created by excess glucose [169].

There are few studies to associate SFN with heart disease but significant cardioprotection was demonstrated in an animal study [126] using fresh broccoli homogenate. Changes included improved postischaemic ventricular function, reduced myocardial infarct size, and decreased cardiomyocyte apoptosis after the rats were sacrificed. These findings correlated with increased levels of Trx as well as HO-1.

A 1997 study [172] investigating the role of thioredoxin in vascular biology describes the induction of mitochondrial antioxidant enzyme, superoxide dismutase (MnSOD) by Trx. In addition, Trx influences hormones such as insulin as well as glucocorticoid receptors and other proteins such as endothelial nitric oxide synthase and signalling proteins such as transcription factors. The findings of a Phase 1 clinical trial [25] demonstrated that 100 grams of fresh broccoli sprouts over a 7-day period provided cardiovascular benefits which included favourable changes in blood lipids as well as reduction in biomarkers of oxidative stress. This study however did not assay the broccoli sprouts for their SFN yield, limiting its usefulness.

### 6.3. NAD(P)H Quinone Dehydrogenase 1: Beyond Redox Modulation

NAD(P)H quinone dehydrogenase 1 (coded by the gene *NQO1* and with the enzyme sometimes abbreviated as NQO1) is emerging as an Nrf2-target enzyme with broad cytoprotective properties. A paper [173] published almost two decades ago claims that *an extensive body of evidence supports the conclusion that catalysing obligatory two-electron reductions of quinones to hydroquinones, NQO1, protects cells against the deleterious effects of redox cycling of quinones and their ability to deplete glutathione.* The same researchers [144] have since published on this topic discussing what they describe as *a* “*multifunctional antioxidant enzyme and exceptionally versatile cytoprotector*.” They suggest too that NQO1 with cytoprotective roles which extend well beyond its catalytic function could be considered as a “*marker cytoprotective enzyme.*” Further, they state that *NQO1 is one of the most consistently and robustly inducible genes among members of the cytoprotective proteins.*

### 6.4. NQO1 Pharmacokinetics following SFN Ingestion

A study used breast tissue to measure the pharmacokinetics of *NQO1* induction over 24 hours, following a single serve of a broccoli sprout homogenate (SFN = 200 *μ*mol) one hour prior to mastectomy [88].

**Figure 8:**
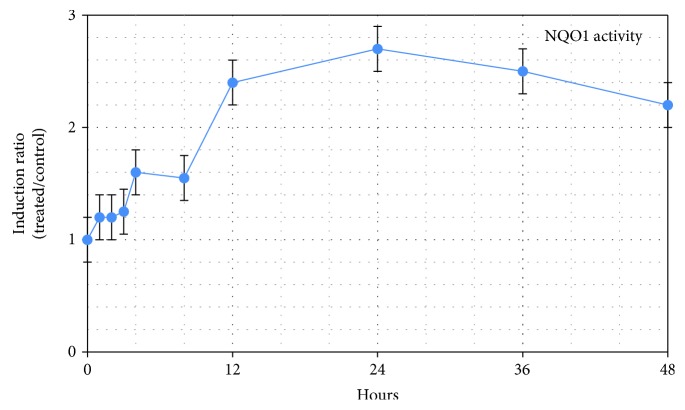
NAD(P)H quinone dehydrogenase 1 activity over time following sulforaphane ingestion (an image from Cornblatt [88]).

Maximal induction of NQO1 occurred at around 24 hours, declining thereafter (Figure 8). This peak represents an approximate 2.8-fold induction over baseline. These findings are useful when considering the effect of SFN as an intervention material in acute compared with chronic conditions. A significant increase in NQO1 occurred between 6 and 12 hours, a timeframe that may not be sufficiently responsive for management of an acute state, leaving one to conclude that NQO1 induction is best suited to chronic conditions where a rapid response may not be necessary.

### 6.5. Comparative Phytochemical NQO1 Induction

The induction of NQO1 has been investigated in different studies to compare the effect of well-known phytochemicals [146, 168, 174]. The comparatively much higher NQO1 induction by SFN against popular plant-derived supplements is evident [146].

It has been claimed here and elsewhere that SFN is the most potent naturally occurring inducer [146, 175] of this enzyme (Figure 6) NQO1's antioxidant capacity extends to scavenging superoxide directly [176], albeit not as efficiently as does SOD.

### 6.6. NQO1: Recycling Cellular Bioactives

NQO1's other functions extend to the maintenance of coenzyme Q 10 and vitamin E in their active reduced forms [144]. Induction of *NQO1* by SFN also coordinately induces [58] genes encoding cellular NADPH-regenerating enzymes such as glucose-6-phosphate dehydrogenase, 6-phosphogluconate dehydrogenase, and malic enzyme. NADPH in turn assists in maintaining GSH in its reduced state. The NQO1 enzyme provides major antioxidant functions by virtue of its two-electron reduction mechanism; this diverts quinones from participating in oxidative recycling and production of ROS and prevents mutagenic changes to DNA [144, 177, 178]. This function is clinically relevant to chemoprevention.

### 6.7. Haemoxygenase-1 (HO-1)

HO-1 is an inducible isoform of the first and rate-controlling enzyme of the degradation of haem into iron, carbon monoxide, and biliverdin, the latter being subsequently converted into bilirubin [179]. HO-1 is considered to have potent cytoprotective effects which include antioxidant and anti-inflammatory properties in cardiovascular and other tissues. It has been suggested that cytoprotection may be due to bilirubin directly inhibiting NADPH oxidase activity, thereby reducing superoxide generation [180].

Although the mechanism for the anti-inflammatory effect of HO-1 has not been fully elucidated, there are known associations between HO-1 and a number of cytokines. The 5′-flanking region of the *HO-1* gene contains binding sites for the transcription factors that regulate inflammation, including NF-*κ*B and activator protein-1 (AP1) [181]. Leukocyte *HO-1* gene expression is significantly lower in patients with and without diabetic microangiopathy compared with control subjects and normalization of blood glucose results in a reduction in HO-1 antigen in the cytoplasm of mononuclear leukocytes [182].

Hyperglycaemia is known to increase the formation of advanced glycation end products (AGEs). In endothelial cells, the interaction of the AGE with its receptor, RAGE, induces generation of ROS, NF-*κ*B translocation, and expression of several proinflammatory and procoagulatory molecules [183]. In normal cells, RAGE is present at low levels but is increased in the endothelia of diabetics [180].

Given the theme of the above discussion, it could be asked whether the redox-inflammation couple could be the common upstream factor at play in a number of chronic diseases, of which T2DM is an example. It has, in fact already been proposed [7, 184] that oxidative stress is the pathogenic mechanism linking insulin resistance with dysfunction of both pancreatic beta-cells and the endothelium, eventually leading to overt diabetes and cardiovascular disease.

### 6.8. Redox Effects in Phase 1 vs. Phase 2 Detoxification Pathways

**Figure 9:**
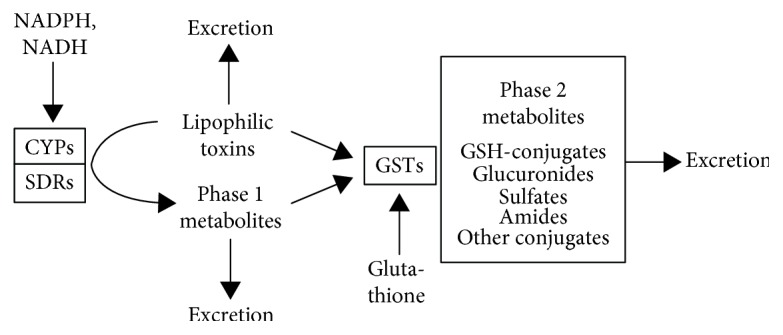
Interaction of Phase 1 and Phase 2 metabolites in detoxification (an mage from McElwee et al. [192]).

As long ago as 1985, it was determined that the ideal chemoprotective compounds are monofunctional inducers of Phase 2 detoxification enzymes. Monofunctional inducers function by metabolising the oxidative and carcinogen-activating products of the Phase 1 enzymes, without having any significant effect on Phase 1 activity itself [185]. Toxins presented to the Phase 1 enzymes produce intermediate compounds which are sometimes more toxic to cells than the initial toxin. It is therefore important that Phase 2 is sufficiently active that the intermediate products cannot accumulate in the cellular environment. The majority of chemical carcinogens require metabolic activation by Phase 1 before they can initiate cancer [186].  Figure 9 illustrates the Phase 1 and Phase 2 detoxification pathways [187].

As a monofunctional inducer, SFN has been described an ideal detoxifier, as its effect on Phase 1 is minimal compared with its significant activity on Phase 2 [188]. By comparison, many of the most potent of the synthetic SFN analogues [189] are bifunctional inducers and not the monofunctional inducers having the most chemopreventive effect. Several synthetic compounds [190] have been investigated for their chemopreventive potential against lung cancer in smokers [191].

The process of cellular detoxification of both exogenous and endogenous factors entails two phases: Phase 1 (oxidative activation reactions) and Phase 2 (conjugative reactions), effected by several large and diverse gene families [192].

### 6.9. Significance of Induction of Phase 1 and Phase 2 Detoxification Enzymes

Not all Brassica-derived compounds are monofunctional inducers. Indole-3-carbinol (I-3-C) derived from the mature broccoli vegetable is a bifunctional inducer and as such may lead to the generation of highly toxic intermediate compounds which may overwhelm the capacity of the localised direct-acting antioxidants to quench them or the Phase 2 processes to detoxify them [193].

By contrast, SFN selectively upregulates Phase 2 detoxification enzymes, minimising the risk of generating excessive amounts of reactive intermediates (Figure 9 [192]). As a consequence, although some I-3-C animal studies show an anticarcinogenic effect, other studies using I-3-C show it to have carcinogenic potential where comparable studies using SFN do not [194–196]. It should be noted that the comparatively small quantity of I-3-C generated from the glucosinolates in broccoli vegetable is unlikely to replicate the effects of isolated synthetic I-3-C concentrations used in cell culture studies [87].

## 7. SFN: Its Anti-Inflammatory Effects

### 7.1. Regulation of NF-*κ*B

Members of the NF-*κ*B family of transcription factors function as dominant regulators of inducible gene expression in virtually all cell types in response to a broad range of stimuli, with particularly important roles in coordinating both innate and adaptive immunities [197], as well as inflammatory responses, cell differentiation, proliferation, and apoptosis.

NF-*κ*B is controlled by various mechanisms of posttranslational modification and subcellular compartmentalisation as well as by interactions with other cofactors or corepressors [198]. The NF-*κ*B family of transcription factors includes RelA (p65), RelB, and others and as a complex, NF-*κ*B mediates immune responses to cellular challenges that include bacterial and viral infection and inflammation [63].

The activity of NF-*κ*B is tightly regulated at multiple levels, a factor that may be associated with its influence on the expression of numerous genes [199]. Nuclear translocation of NF-*κ*B is primarily controlled by signalling associated with I*κ*B kinase (IKK) in two related pathways associated respectively with the NF-*κ*B classical (canonical) and alternative pathways.

Among the most potent NF-*κ*B activators are tumour necrosis factor (TNF-*α*), interleukin (IL)-1*β* and bacterial lipopolysaccharide (LPS), with TNF-*α* activation being one of the best characterised of the NF-*κ*B signalling pathways [200].

### 7.2. The Action of NF-*κ*B in Intestinal Epithelial Cells

Nrf2 and NF-*κ*B are both well-studied cellular transcription factors, and their effects occur in all cells including those of the intestinal epithelium. The gut-immune interface describes the signalling network that connects the intestinal epithelial cells to the immune cells of the lamina propria, situated directly below the epithelium [201]. Here, the microbiota, via this interface, also influence immune function including inflammatory pathways. As such, the gut-immune interface directly connects the cellular functions of redox-balance, inflammation, and infection control via immune modulation.

### 7.3. SFN at the Gut-Immune Interface

Because SFN has been shown to inhibit NF-*κ*B in endothelial cells [202], it is likely the same effect would occur in other epithelial cells such as the intestinal epithelium, thereby retarding local inflammation.

Whereas SFN directly activates cytosolic Nrf2, its action on NF-*κ*B is to inhibit NF-*κ*B binding to the DNA [203]. NF-*κ*B plays a key role in the immune system where it is activated by a series of events initiated by Toll-like receptors (TLR) on epithelial cells [204]. TLR2 and TLR4 can identify distinct molecular patterns on the cell wall of invading pathogens. These patterns act as innate sensors but also shape and bridge innate and adaptive immune responses.

### 7.4. Cross Talk between Nrf2 and NF-*κ*B

SFN is associated with cellular defences via mechanisms governed by the transcription factors Nrf2 and NF-*κ*B; molecular cross talk between these transcription factors has been reported [63]. Imbalance between Nrf2 and NF-*κ*B is associated with a significant number of diseases across various body systems, and these relationships are the subject of extensive research in cancer biology in particular [205].

Although the complex interplay between Nrf2 and NF-*κ*B has been highlighted, there remains much to be explored in order to understand how such relationships may impact disease pathophysiology at the molecular level. As part of the cross talk between these two transcription factors, NF-*κ*B has been shown to regulate Nrf2-mediated ARE expression. Several mechanisms exist by which p65 (the canonical NF-*κ*B subunit) can exert negative effects on ARE-linked gene expression [206]. It would seem that the cross talk between Nrf2 and NF-*κ*B enables cells to more finely regulate their responses to cellular stressors.

### 7.5. Immune Modulation (Anti-Inflammatory Effects)

Activation of TLR4 by the endotoxin released by gram-negative bacteria results in signalling that activates NF-*κ*B with subsequent generation of inflammatory cytokines [204]. Toll-like receptor (TLR4) pathways mediate proinflammatory cytokine and interferon responses [207]. SFN has been shown in a thiol-dependent manner to suppress TLR4 oligomerization. Saturated fatty acids are known to act as ligands for TLR4 in macrophages and adipocytes, with these signals in turn regulating various proinflammatory transcription factors [208]. More recently, in-depth investigation of the microbiome has uncovered the pathways that link these very signals to cardiometabolic effects, thereby connecting the gut-immune relationship to systemic disease [13].

### 7.6. Effect of SFN on Inflammation Markers in Humans

In a recent study using 30 grams of fresh broccoli sprouts incorporated daily into the diet, two key inflammatory cytokines were measured at four time points in forty healthy overweight people [209]. The levels of both interleukin-6 (Il-6) and C-reactive protein (CRP) declined over the 70 days during which the sprouts were ingested. These biomarkers were measured again at day 90, wherein it was found that Il-6 continued to decline, whereas CRP climbed again. When the final measurement was taken at day 160, CRP, although climbing, had not returned to its baseline value. Il-6 remained significantly below the baseline level at day 160.

**Figure 10:**
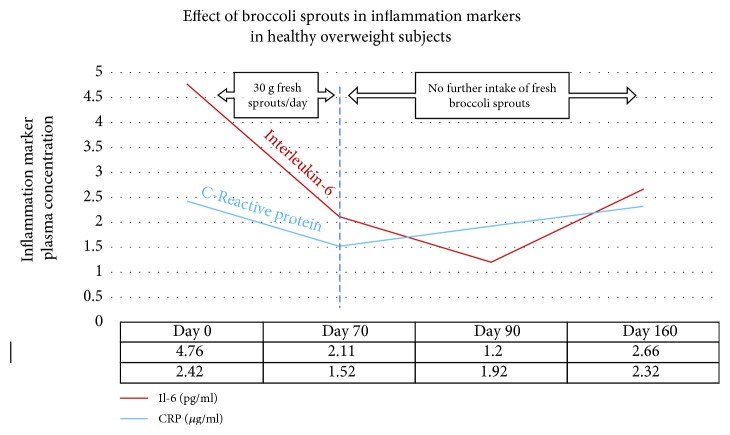
Effect of broccoli sprouts in inflammation markers in healthy overweight subjects (data from Lopez-Chillon et al. [209]).

The sprouts contained approximately 51 mg (117 *μ*mol) GRN, and plasma and urinary SFN metabolites were measured to confirm that SFN had been produced when the sprouts were ingested. The data from this study are expressed visually in Figure 10.

### 7.7. Effect of SFN on Inflammation Markers in Type 2 Diabetes Patients

Where the study described above by Lopez-Chillon et al. investigated healthy overweight people to assess the effects of SFN-yielding broccoli sprout homogenate on biomarkers of inflammation, Mirmiran et al. in 2012 had used a SFN-yielding supplement in T2DM patients [210]. Although the data are not directly comparable, the latter study using the powdered supplement resulted in significant lowering of Il-6, hs-CRP, and TNF-*α* over just 4 weeks. It is not possible to further compare the two studies due to the vastly different time periods over which each was conducted.

## 8. SFN: Its Antimicrobial Effects

The complex signalling mechanisms discussed above will apply in a general sense to the modulation of core upstream processes that occur in human cells in general. In the following section, specific actions by SFN exhibit an antimicrobial effect on a common gut pathogen. It is not known at this stage whether the mechanisms are applicable to eradication of other pathogens with similar characteristics.

### 8.1. SFN and Helicobacter pylori Gut Infection

Although a direct antimicrobial effect has been demonstrated for extracts of cruciferous vegetables [211], the effect is not considered clinically relevant. More recently, SFN has been shown mechanistically and clinically to have a direct bactericidal effect on the *Helicobacter pylori* bacterium via two separate mechanisms.

Approximately half of the global population is thought to be colonised by the *H. pylori* organism, making its classification as either a pathogen or a commensal uncertain; as such, it is sometimes described as a pathobiont [212]. *H. pylori* is shown to be symptomatic in some people and not in others, indicating that there may be individual control mechanisms that keep the organism in check. The popular dietary practice of salting food can also contribute to its pathogenicity. Sodium chloride, in the presence of *H. pylori*, becomes a cancer promoter, enhancing chronic gastric mucosal membrane inflammation [213].

*H. pylori* infection may be asymptomatic but by raising the pH of the gastric contents via continuous synthesis of ammonia, it contributes to impaired protein digestion and macromineral malabsorption. Iron absorption is well known to be impaired in the presence of *H. pylori* [214].

Consideration of the *upstream* processes that cells use to maintain homeostasis might indicate that the redox-inflammation couple might be associated. Recently, Yanaka, who had undertaken some of the earlier *H. pylori* trials using SFN as an intervention, reviewed several of the mechanisms by which Nrf2 activators may exhibit their antimicrobial effect [215]. Yanaka argues that significant protection to the gastrointestinal tract is afforded by the modulation of oxidative stress and inflammation as a result of simultaneous activation of Nrf2 and downregulation of NF-*κ*B [216].

In their 2009 study, Yanaka et al. demonstrated that broccoli sprouts suppressed the upregulation of the inflammatory markers, TNF-*α* and IL-1*β* in the gastric mucosa by *H. pylori* infection in a wild type but not in Nrf2−/− mice, suggesting a systemic protective effect against gastritis that was the result of Nrf2 activation [217].

Over the past fifteen years, two clinical trials have demonstrated SFN's bactericidal effect on the *H. pylori* organism, a bacterium which is associated with gastric reflux and cancer [217, 218]. In the initial study, forty-eight *H pylori*-infected subjects were given 70 grams fresh broccoli sprouts daily [217]. Three markers of *H. pylori* infection declined within eight weeks to below the diagnostic cutoff point. However, once the intervention had stopped, the levels of *H pylori* returning to baseline levels after 8 weeks.

### 8.2. Urease Inhibition as a Mechanism for Regulating H. pylori Colonies

In extending the earlier SFN—*H. pylori* research, the urease-positive nature of the *H. pylori* gram-negative bacterium has been investigated. Urease activity in human and animal cells can be the cause of some pathogen-induced infections, and the ongoing quest to provide appropriate urease inhibitors includes the search for natural sources [219]. SFN has been demonstrated to exhibit urease activity, thereby potentially providing a clinical alternative to pharmaceutical antibiotics to control *H. pylori* gastric infections [220].

**Figure 11:**
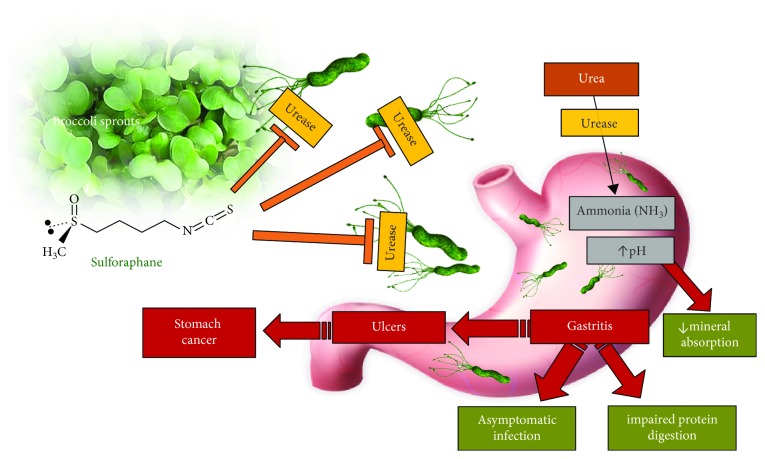
The proposed mechanism by which SFN inhibits urease synthesis by H. pylori and, in so doing, reduces the disease risks associated with H. pylori infection (an image adapted from Fahey et al. [220]).

It is known that *H. pylori* uses urease to hydrolyse protein-derived urea available in the human gut lumen in order to synthesise ammonia; up to 10% of the total protein content of the *H. pylori* organism comprises the urease enzyme [221]. The presence of urease is also essential to enable *H. pylori* to colonise the gastric mucosa [222]. This results in partial neutralisation of the low gastric pH. The resultant elevated pH provides the preferred conditions that enable *H. pylori* to thrive (Figure 11). Several pH-sensitive urease inhibitors of varying potency have been identified, and these include ammonia (the product of urease on its substrate), thiols, sulfite, fluoride, green tea-derived epigallocatechin gallate (EGCG), and heavy metals [222].

*H. pylori* is not the only colonising microbe with urease activity. According to Auron and Brophy in 2012, ureolytic microbes in the digestive or urinary tracts potentially contribute to hepatic encephalopathy and coma, resulting in hyperammonaemia and brain intoxication [223]. Several other potentially pathogenic urease-positive microbes have been identified; *Klebsiella aerogenes*, *Brevibacterium ammoniagenes*, *Morganella morganii*, *Proteus mirabilis*, *Staphylococcus saprophyticus*, *Escherichia coli*, *Yersinia enterocolitica*, and *Haemophilus influenzae* are among the better-known [222]. Whether SFN is capable of reducing their virulence in humans by urease inhibition is not yet known.

## 9. Phytochemicals on the Drug Discovery Path

### 9.1. How do Clinical Trial Data Inform Dose?

For a phytochemical to be considered as a therapeutic agent, it must be evaluated using many of the same tools used in pharmaceutical product development. Whereas a pharmaceutical is typically a single molecule, plants are complex multicomponent mixtures; the phytochemical composition of which is not constant due to factors which include inherent agricultural and environmental variability [224].

Of the published SFN research to date, the intervention materials are nonstandard, with some studies using the pure chemical SFN as the intervention material where others use broccoli vegetable, fresh or dried broccoli sprouts; therefore, comparison of clinical trial outcomes becomes more difficult.

Nevertheless, when working with isolated bioactive phytochemicals and whole foods as a source of the same bioactive, the biopharmaceutical processes typically used in pharmaceutical development should equally apply. The LADME principles (liberation, absorption, distribution, metabolism, and excretion) described in connection with the pharmacokinetics of pharmaceuticals should be equally relevant to phytochemicals [225]. However, such data is seldom available for the more popular phytochemicals used preventively or medicinally [224]. A comprehensive review on this subject by Pferschy-Wenzig and Bauer [224] highlights the many issues that can be underappreciated by consumers who self-medicate on the basis of limited safety and efficacy data.

The literature for SFN indicates that many researchers have addressed the various LADME principles, thereby producing a more extensive database that is useful for interpreting the dose-response.

### 9.2. Published Clinical Trials

There are currently over 1900 published papers which appear in a PubMed search using the term, “sulforaphane” (PubMed accessed February 4^th^, 2019). However, there is a limited number of clinical trials utilising either fresh or processed broccoli sprouts (Table 3). Not all trials quantitatively specify the bioactive content of the intervention material. As a result, it is difficult to interpret their findings in a clinical context.

Table 3 illustrates the range of SFN doses used in selected clinical trials where the endpoint is a common human disease or a disease biomarker. Although these trials are of short duration and with small numbers of participants, these data enhance our understanding of the potential of SFN as a clinical intervention. Although dose forms and study populations and endpoints are different across the selected trials, a pattern emerges to show that clinical outcomes are achievable in conditions such as asthma [226] with daily SFN doses of around 18 mg daily and from 27 to 40 mg in type 2 diabetes [24, 227].

A lower SFN dose of around 9-14 mg daily yielded a positive outcome in the autism study by Singh et al. [228], whereas *H. pylori* control was effective with a higher dose of 30 mg SFN daily. Of the available trials, the prostate-specific antigen (PSA) doubling time after radical prostatectomy selected the higher 60 mg daily dose [229].

In considering SFN as a therapeutic intervention, some important questions to be asked are as follows: “What quantity of starting material is needed to achieve a micromolar concentration which generates a significant clinical outcome?” “How can a broccoli sprout raw material be produced which will be consistent in its composition?” and “Is it possible to produce a broccoli sprout raw material that is a practical solution to consumer needs for a SFN-yielding supplement?”

### 9.3. The Clinician's Dilemma in Applying Clinical Trial Data

Unlike products categorised by U.S. law as “*dietary supplements*,” the subgroups of products claiming to be “*nutraceutical supplements*” are typically standardised for their bioactivity; this may require that one or more bioactives are specified with each batch produced. Of the various available supplements which list a dried broccoli sprout or seed ingredient, the label disclosure is both inconsistent and misleading. Products labelled as “extracts” are manufactured such that GRN is retained as the extract and the myrosinase enzyme needed to synthesise SFN from its glucoraphanin precursor is inactivated [54].

A consumer or a clinician intending to select an available SFN-yielding supplement on the basis of its dose compared with those used in the peer-reviewed published clinical trials has, until very recently, had great difficulty in doing so, given that sprout and seed “extracts” are typically labelled as containing “sulforaphane glucosinolate,” a descriptive commercial name that refers to “glucoraphanin” [236]. Some conversion of GRN to SFN can occur in response to metabolism by the gut microflora; however, the response is inefficient, having been shown to vary “from about 1% to more than 40% of the dose” [237].

Standardisation of label disclosure to remove inconsistency and ambiguity would greatly assist both clinicians and consumers in determining the appropriate daily dose needed to match the doses used in the clinical trials [236].

### 9.4. Addressing a Conundrum

Because SFN is derived from a commonly consumed vegetable, it is generally considered to lack adverse effects; the safety of broccoli sprouts has been confirmed [238]. Furthermore, a 2018 publication concluded as follows: *“it is clear that SFN is a safe and relatively nontoxic chemopreventive agent and exerts anticancer activities through multiple mechanisms, including regulation of Phase I and Phase II drug-metabolising enzymes, anti-inflammatory activity, cell cycle arrest, induction of apoptosis, and the epigenetic regulation on Nrf2-Keap1, cyclins, and CDK”* [239].

However, the use of a phytochemical in chemoprevention engages very different biochemical processes when using the same molecule in chemotherapy; the biochemical behaviour of cancer cells and normal cells is very different [240]. As such, it cannot be assumed that SFN as a chemopreventive can be appropriately utilised in the context of chemotherapy where active cancer has been diagnosed.

No discussion of SFN and Nrf2 would be complete without reference to the fact that both Nrf2 activators and Nrf2 inhibitors can be utilised in cancer therapy [241–243]. Cancer cells are able to hijack the Keap1-Nrf2 system via multiple mechanisms leading to enhanced chemo- and radioresistance and proliferation via metabolic reprogramming as well as inhibition of apoptosis [241]. One such mechanism is associated with stimulating the coordinated induction of hepatic Multidrug Resistance Proteins (MRPs) which are adenosine triphosphate-dependent transporters that efflux chemicals out of cells. This ATP-binding cassette family of Phase III detoxification transporters (ABC transporters) [244] is involved in the efflux of numerous endogenous and exogenous chemicals, including chemotherapeutic drugs. MRPs play a key role in cellular protection by removing xenobiotics, metabolites, and endogenous substrates that can accumulate in tissues and lead to toxicity. The activation of the Nrf2 regulatory pathway stimulates the coordinated induction of hepatic MRPs, such that the effective dose of the drug is reduced [245]; this may include chemotherapeutic drugs.

A clinician may then ask whether it is prudent to consider therapies that activate Nrf2 in the context of a cancer diagnosis. James Watson, well-known 1962 Nobel Laureate [246], more recently [247] expressed his concerns about the potential risks associated with the use of antioxidant therapy in promoting cancer progression. Watson questions whether free radical-destroying antioxidant nutritional supplements may have caused more cancers than they have prevented [247].

In the same year that Watson published his viewpoint, Sporn and Liby suggested that, aside from the extensive literature on the suppression of carcinogenesis by Nrf2 activation, conversely this transcription factor may be oncogenic and cause resistance to chemotherapy [115]. Their opinion article, they say, is aimed at rationalising these conflicting perspectives by critiquing the context dependence of Nrf2 functions and the experimental methods behind these conflicting data. An important new concern they suggest is the finding that common oncogenes, such as *KRAS*, *BRAF*, and *MYC*, all increase the transcription and activity of NRF2, resulting in an increase in cytoprotective activity within the cancer cell [115]. As well, they query the possible effects of Nrf2 polymorphisms, suggesting that enhancement of *NRF2* activity (caused by mutations) can protect tumours from the cytotoxic effects of reactive oxygen species that are induced by chemotherapy or that may be produced endogenously by oncogenic signalling in advanced tumours.

They conclude and rationalise by suggesting that the effect of Nrf2 activation is largely related to the biological time context, stating that Nrf2 activity is desirable (for the host organism) in early stages of tumourigenesis, when the host is seeking to control premalignant carcinogenesis, but is undesirable in later stages of tumourigenesis, when it could make fully malignant cancer cells become resistant to treatment.

A very recent paper [112] highlights this dual role and its implications for Nrf2 activation. It suggests that because Nrf2 can modulate the detoxification pathways, its effect on anticancer drugs may lead to chemoresistance and that the switch between a beneficial and a detrimental role for Nrf2 in cancer cells depends on a number of factors which include the tight control of its activity. This poses an obvious dilemma which is already under active discussion and investigation [113, 115]; SFN and other phytochemicals capable of modulating Nrf2 form part of such investigation [112].

Until this dilemma is resolved, clinicians recommending nutraceutical supplements would be wise to avoid coadministration of any nutraceutical supplement whilst the patient is undergoing chemotherapy. Even though “Principles of Care Guidelines” are promoted by an organisation representing such clinicians, it seems clear from the aforegoing discussion that there remains insufficient evidence for coadministration of supplements during oncotherapy [248].

That aside, a different line of investigative research has considered whether a role exists for phytochemicals to be utilised in conjunction with chemotherapy. A number of in vitro studies using different cancer cell lines have investigated the potential for SFN (via several mechanisms) to be utilised in conjunction with chemotherapeutic drugs. The goal of such studies is to enable higher doses of the drug to be used before reaching the toxicity threshold of the normal cells [249–255].

In one study as an example of this process, SFN was shown to reduce the toxicity of the chemotherapeutic drug cadmium selenide (CdSe) in human hepatocytes by induction of GSH synthesis at concentrations of 2.5, 5.0, and 10.0 *μ*M SFN, thereby protecting the liver against cytotoxicity and enabling a higher chemotherapeutic dose to be used [250]. Here, SFN's effect on GSH concentration exhibited a linear dose-response; however, it is unlikely that the higher concentration could be achieved clinically using either diet-derived or supplemental SFN.

Clearly, there is much to be learned before phytochemicals including SFN [256] can be recommended for patients with diagnosed cancers, whether or not the patient is undergoing chemotherapy. Even so, the case for the disease-preventive (including chemopreventive) effects of cruciferous vegetable consumption in general is strong [257, 258] and the last twenty-seven years have witnessed a growing body of evidence to support the roles of SFN in disease prevention, especially given its superior potency as a highly bioavailable Nrf2 activator [259–264].

Albeit limited, the available SFN clinical trial data indicate positive outcomes for a number of common human conditions for which the SFN doses are known. Perhaps future research will more closely focus on its potential effects in patients with diagnosed cancer with a view to resolving the current conundrum.

## 10. Conclusion

Many decades of research have established strong links between cellular redox and immune imbalances, and the development of chronic disease and biomarkers associated with oxidative stress and inflammation have verified the relationship. However, several large-scale clinical trials to prevent diseases such as T2DM, CVD, and cancer with antioxidant vitamin supplements failed to demonstrate the expected prevention and, in some cases, led to worsening of the biomarkers.

It was not until the discovery of the transcription factor, Nrf2, in 1994 that it became clear that although an enhanced redox balance within the cells was required, the antioxidant vitamins were unable to deliver this. As the understanding of nutrigenomic principles evolved, it became clear that plants contained bioactive phytochemicals that were capable of activating Nrf2; this resulted in the induction of gene expression that targeted a large battery of the genes associated with the core *upstream* cellular defence processes. A key advantage of using phytochemicals to target Nrf2 *upstream* is that a potent Nrf2 activator is capable of inducing hundreds of genes simultaneously.

Of the phytochemicals with Nrf2 inducer capacity, Brassica-derived SFN is the most potent naturally occurring biomolecule known at this time. It is not only a potent Nrf2 inducer but also highly bioavailable so that modest practical doses can produce significant clinical responses. The daily SFN dose found to achieve beneficial outcomes in most of the available clinical trials is around 20-40 mg. With a potent, myrosinase-active whole broccoli sprout supplement, these doses can be attained with just a few capsules daily.

Other Nrf2 activators such as shown in Figure 6 not only lack potency but also lack the bioavailability to be considered as significant intracellular Nrf2 activators. Our understanding of the roles for poorly bioavailable polyphenols in human health is evolving to one more associated with its interactions with the microbiota and the uncertain functions of the metabolites generated by the microbes [55].

Although most of the research on SFN is associated with its ability to activate Nrf2, it exhibits a range of other effects. This review has discussed the way in which another transcription factor, NF-*κ*B, which is associated with inflammatory pathways is downregulated by SFN. This dual action of SFN is especially intriguing in that Nrf2 and NF-*κ*B interact via their own “cross talk”.

Infection control is another key activity of the immune system and is closely associated with NF-*κ*B. In this vein, SFN has been shown to inhibit the *H. pylori* bacterium, a significant gastric cancer risk factor that is prevalent globally. Since pharmaceutical solutions to *H. pylori* eradication are only partially and temporarily effective, the need for a safe, effective therapy is pressing. SFN has been found to inhibit and may possibly even eradicate *H. pylori* in humans via two separate mechanisms.

This review has explored the issues associated with the development of a nutraceutical supplement with significant ability to beneficially influence many of the *upstream* processes associated with core cellular defences; SFN emerges as a potential candidate of this class. The available dose-response evidence is promising, albeit limited so that larger clinical trials will clearly be needed. Even so, the existing data reveal a dose-response that appear to be reasonably consistent by disease state and tissue type and that doses of around 20-40 mg SFN daily can be provided in practical dose form quantities. SFN's primary advantage over many other phytochemicals lies in its comparatively high bioavailability together with its capacity to potently induce Nrf2 target genes.

Has SFN *come of age* as a clinically relevant nutraceutical in the prevention and treatment of chronic disease? Perhaps not just yet; however, the continuing interest in this somewhat novel phytochemical shows no sign of slowing.

## Figures and Tables

**Table 1:** Major randomized placebo-controlled trials investigating the effects of the antioxidant supplement on prevention of diabetes or glucose homeostasis.

Study	Study population	Duration (years)	Antioxidants (daily dose)	Endpoint	Results
Women's Health Study	38,716 healthy U.S. women	10	*Vitamin E* (*α*-tocopherol: 600 IU; 933.3 *μ*mol)	Incident diabetes	No effect

Women's Antioxidant Cardiovascular Study	6,574 nondiabetic U.S. women at high risk of cardiovascular disease	9.2	*Vitamin E* (*α*-tocopherol: 300 IU; 466.7 *μ*mol) *Vitamin C* (500 mg; 2.84 Mmol) *Beta-carotene* (25 mg; 46.6 *μ*mol)	Incident diabetes	No effect

Physician Health Study	22,071 healthy U.S. male physicians	12	*Beta-carotene* (25 mg; 46.6 *μ*mol)	Incident diabetes	No effect

Alpha-Tocopherol, Beta-Carotene Cancer Prevention Study	27,379 nondiabetic male Finnish smokers	12.5	*Vitamin E* (*α*-tocopherol 50 mg; 116.1 *μ*mol) *Beta-carotene* (20 mg; 37.3 *μ*mol)	Incident diabetes	No effect

Supplementation with Antioxidant Vitamins and Minerals study	3,146 nondiabetic French	7.5	*Vitamin C* (120 mg; 681.4 *μ*mol) *Vitamin E* (30 mg; 104.5 *μ*mol) *Beta-carotene* (6 mg; 11.2 *μ*mol) *Selenium* (100 *μ*g; 1.27 *μ*mol) *Zinc* (20 mg; 306 *μ*mol)	Fasting glucose	No effect

**Table 2:** Summary of clinically relevant actions of SFN.

Action	Clinical implications
(1) Increases synthesis of glutathione [117].	This has implications for oxidative stress and detoxification as glutathione is the substrate for both pathways. Glutathione is also an antioxidant in its own right.

(2) Inhibits some Phase 1 detoxification enzymes that activate chemical carcinogens [118].	This reduces the level of toxic intermediates with carcinogenic potential. It also allows Phase 2 to “keep pace” with Phase 1 processing.

(3) Increases activity of Phase 2 detoxification enzymes. Sulforaphane is considered the most potent of the Phase 2 inducing substances [79].	As a monofunctional inducer, sulforaphane is considered to be a significant component of the anticarcinogenic action of broccoli.

(4) Provides significant antioxidant activity, largely due to its ability to induce glutathione synthesis.	Glutathione is a critical factor in protecting organisms against toxicity and disease [119]. The ability of sulforaphane to upregulate glutathione synthesis is highly significant.

(5) Acts as a histone deacetylase inhibitor, providing DNA protection [120–122].	Development of histone deacetylase inhibitors is a key avenue for cancer drug research.

(6) Induces apoptosis, inhibits MMP-2 (metastasis), and inhibits angiogenesis and cell cycle arrest [28, 105, 123, 124] (interacts at several levels).	Therapeutic interventions which exhibit several related actions targeting the same underlying defect are considered highly desirable.

(7) Limits proinflammatory effects of diesel chemicals by upregulation of Phase 2 enzymes [125].	Environmental pollutants are known to contribute to various lung diseases. Removal of the toxins reduces tendency to disease.

(8) Induces thioredoxin (Trx) as part of the ARE.	Thioredoxin is implicated in cardioprotection by triggering several *survival* proteins [126]. Sulforaphane may have beneficial effects in cardiovascular disease.

(9) Bactericidal against *Helicobacter pylori* and also blocks gastric tumour formation in animals [127].	Helicobacter is known to contribute to development of stomach cancer. Elimination of the organism without the use of typical antimicrobial *Triple Therapy* could protect the colonic microflora.

(10) Protects dopaminergic cells from cytotoxicity and subsequent neuronal death (cell culture) [128].	Dopaminergic neurones are associated with Parkinson's disease. Pharmaceuticals to treat Parkinsonism are not without risk and the disease is not usually detected until more than 50% of the neurones have been lost. A chemoprotective tool could prevent premature loss.

(11) Increases p-53 (associated with tumour suppression) and bax protein expression, thereby enhancing cellular protection against cancer [129].	Sulforaphane is an attractive chemotherapeutic agent for tumours with a p53 mutation [62].

(12) Limits effect of aflatoxin on liver cells [26].	Interventions which can offer significant protection against environmental and food-borne pollutants could prevent the consequences of these factors. Appropriate doses of sulforaphane-yielding substances are yet to be determined.

(13) Enhances natural killer cell activity and other markers of enhanced immune function [117].	The immune system is a critical part of the body's defences against inflammatory as well as infectious diseases. Most diseases benefit from enhancement to immune function.

(14) Suppresses NF-*κ*B, a key regulator of inflammation [117]. NF-*κ*B expression is downregulated by sulforaphane and as such downregulates inducible proinflammatory enzymes such as cyclooxygenase (COX-2) and NO synthase (iNOS).	As an inhibitor of NF-*κ*B as well as an activator of Nrf2, SF modulates many cancer-related events, including susceptibility to carcinogens, cell death, cell cycle, angiogenesis, invasion, and metastasis [117].

(15) Sulforaphane is not directly antioxidant. Instead, it exhibits a weak *prooxidant* effect [130].	Because sulforaphane is not directly antioxidant but exerts its antioxidant effect primarily by induction of glutathione and other antioxidant compounds, it is considered to exhibit an *indirect* antioxidant effect.

(16) Potent inducer of HO-1 (haemoxygenase-1).	Haemoxygenase-1 plays an important role in modulating the effects of oxidants in the lungs [131].

**Table 3:** Sulforaphane dosage from lowest to highest in selected clinical trials.

Condition	~Daily SFN dose	First author	Year
Equivalent sulforaphane dose			
Autism	9-14 mg (50.8–79.0 *μ*mol)	Singh et al. [228]	2014
Nasal allergic response	18 mg (101.5 *μ*mol)	Heber et al. [230]	2014
Asthma	18 mg (101.5 *μ*mol)	Brown et al. [226]	2015
Chronic obstructive lung disease	19 mg (107.2 *μ*mol)	Riedl et al. [29]	2009
Helicobacter pylori infection	30 mg (169.2 *μ*mol)	Yanaka et al. [217]	2009
Gastric mucosal repair	30 mg (169.2 *μ*mol)	Yanaka A. [216]	2011
Detoxification (atmospheric pollution)	36 mg (203.0 *μ*mol)	Egner et al. [231]	2011
Type 2 diabetes	40 mg (225.6 *μ*mol)	Bahadoran et al. [232, 233]	2012
Prostate-specific antigen (PSA) doubling time	60 mg (338.4 *μ*mol)	Cipolla et al. [229]	2015
FRESH BROCCOLI SPROUTS			
*Helicobacter pylori* infection	14-56 grams of fresh sprouts	Galan et al. [218]	2006
Inflammation markers in overweight	30 grams of fresh sprouts	Lopez-Chillon [209]	2018
Metabolic syndrome	100 grams of fresh sprouts	Murashima et al. [25]	2004
Glucoraphanin as myrosinase-inactive broccoli “extract”			
No prevention with 6 pills branded “extract”	180 mg (0.41 mmol) GRN—not SFN	Atwell et al. [234]	2015
Sulforaphane supplement—a null response trial			
*Helicobacter pylori* infection	2 mg (11.28 *μ*mol)	Chang et al. [235]	2015
